# Differential impact of simultaneous migration on coevolving hosts and parasites

**DOI:** 10.1186/1471-2148-7-1

**Published:** 2007-01-10

**Authors:** Andrew D Morgan, Michael A Brockhurst, Laura DC Lopez-Pascua, Csaba Pal, Angus Buckling

**Affiliations:** 1 Department of Zoology, University of Oxford, Oxford, UK; 2 School of Biological Sciences, University of Liverpool, Liverpool, UK; 3 Department of Biology, Indiana University, Bloomington, Indiana, USA

## Abstract

**Background:**

The dynamics of antagonistic host-parasite coevolution are believed to be crucially dependent on the rate of migration between populations. We addressed how the rate of simultaneous migration of host and parasite affected resistance and infectivity evolution of coevolving meta-populations of the bacterium *Pseudomonas fluorescens *and a viral parasite (bacteriophage). The increase in genetic variation resulting from small amounts of migration is expected to increase rates of adaptation of both host and parasite. However, previous studies suggest phages should benefit more from migration than bacteria; because in the absence of migration, phages are more genetically limited and have a lower evolutionary potential compared to the bacteria.

**Results:**

The results supported the hypothesis: migration increased the resistance of bacteria to their local (sympatric) hosts. Moreover, migration benefited phages more than hosts with respect to 'global' (measured with respect to the whole range of migration regimes) patterns of resistance and infectivity, because of the differential evolutionary responses of bacteria and phage to different migration regimes. Specifically, we found bacterial global resistance peaked at intermediate rates of migration, whereas phage global infectivity plateaued when migration rates were greater than zero.

**Conclusion:**

These results suggest that simultaneous migration of hosts and parasites can dramatically affect the interaction of host and parasite. More specifically, the organism with the lower evolutionary potential may gain the greater evolutionary advantage from migration.

## Background

Antagonistic host-parasite coevolution, the reciprocal evolution of host defence and parasite counter-defence, can drive patterns of biodiversity [1-5], host and parasite population dynamics [6], the evolution of parasite virulence [7,8], and may impose selection for the maintenance of sexual reproduction [9]. Recent theoretical work suggests that the dynamics and consequences of host-parasite coevolution depend critically upon the extent to which natural populations are made up of spatially and genetically distinct sub-populations [10-17]. Here, we address how the extent of simultaneous migration of hosts and parasites between sub-populations affects the evolution of host resistance and parasite infectivity in coevolving populations of microbes.

Recent theoretical studies suggest that the relative migration rate of host and parasites is an important determinant of which species will be ahead in a coevolutionary arms race. Migration can increase within-patch genetic variation and hence the rate of adaptation [10-12,15,17]. All other variables being equal, the species that migrates the most is therefore likely to have an evolutionary advantage. (Note, however, that if migration rates are too high, genetic variation may be reduced through reduction of between patch variation, and hence local adaptation; potentially retarding the rate of adaptation [10]). An evolutionary advantage in the case of the parasite is likely to result in increased levels of parasite infectivity, parasite local adaptation (the higher performance of local versus foreign parasites on local hosts), or both. Similar results are expected with respect to host resistance, if hosts migrate more than parasites.

There are examples where parasites and hosts are likely to disperse at different rates (e.g. [18,11]) but in many cases parasite migration is likely to be intimately linked to host migration. If hosts and parasite show the same evolutionary response to migration, and migrate at the same rate, there will be no net effect on average levels of resistance and infectivity, or local adaptation [10]. However, migration will have less impact on the evolvability of a species if it already has greater within-patch genetic variation (e.g. by higher mutation rates or population sizes) or stronger selection for resistance or infectivity, than the other species [17]. Such an asymmetry has recently been demonstrated in our previous work with coevolving populations of the bacterium *Pseudomonas fluorescens *SBW25 and an associated bacteriophage (SBW25Φ 2). In the absence of migration, phages had an evolutionary disadvantage, as demonstrated by phages being locally maladapted to their hosts. Migration of phages alone resulted in a significant increase in phage local adaptation, whereas migration of bacteria alone did not alter patterns of local adaptation relative to unmigrated populations [17]. The mechanism responsible for this asymmetry is unclear, but the smaller average population sizes (2–3 orders of magnitude lower than bacteria) and smaller genome size of phages (40 kb compared with 5 mb), suggests that within-patch genetic variation is likely to be lower for infectivity compared with resistance traits.

In this study, we experimentally address the impact of simultaneous migration of bacteria and phages (*P. fluorescens *SBW25 and SBW25φ2) on the evolution of bacterial resistance and phage infectivity, under a range of migration rates. We hypothesise that phages will benefit more than bacteria from simultaneous migration, as they are more genetically limited, thus having a lower evolutionary potential; and so will gain more from migration. We consider only the early stages of coevolution, where, in this system, bacteria and phages evolve to become increasingly more generalised through time, with respect to their resistance and infectivity; phages evolve to infect a wider range of bacterial genotypes, and bacteria evolve to resist a wider range of phage genotypes [19]. This situation somewhat resembles a Gene for Gene Model (GFGM) of coevolutionary dynamics. Under a pure GFGM, generalists will be favoured, and hence there will be no local adaptation [15]. In natural systems there are likely to be small costs of resistance and infectivity, which drives the interaction to a more Matching Alleles Model (MAM) type of interaction [20]. Under a pure MAM, a precise interaction between parasite and host is needed for successful infection [21]. A MAM type of interaction will therefore favour local adaptation, as each parasite will only be able to infect a certain host genotype [15]. However local adaptation of either bacteria or phages is not observed at these early stages of coevolution in this system despite favourable conditions for its occurrence [22], hence we do not use local adaptation as a measure of the evolutionary advantage of one species or another. Instead, we determine which species has an evolutionary advantage by measuring the resistance of bacteria to their sympatric phages: greater evolutionary potential of phages should result in lower sympatric resistance. In addition to determining how different migration regimes affect the sympatric interaction between bacteria and phages, we also determine how different migration rates affect 'global' levels of bacterial resistance and phage infectivity; i.e. resistance and infectivity across metapopulations. This is biologically meaningful if there is any larger scale migration; i.e. between metapopulations. We quantify these by determining average bacterial resistance to phages that have evolved under all migration regimes, and vice versa for phage infectivity. As above, we hypothesise that migration should provide a greater global benefit to phages than bacteria.

## Results & Discussion

**Figure 1:**
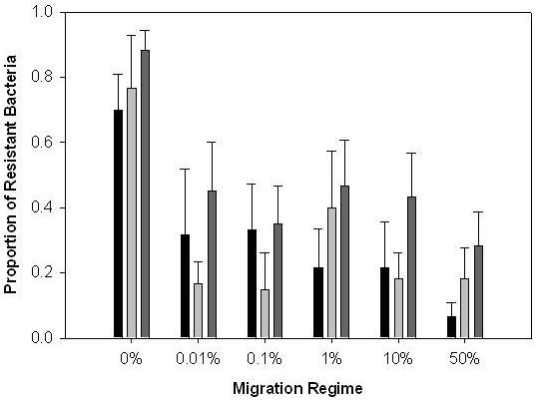
The effect of migration rate on bacterial resistance to their sympatric phages through time. Bars show mean (± 1 SEM) proportion resistant bacteria. Black bars are transfer (time point) 4; light grey bars, transfer 8; dark grey bars, transfer 12.

In this study we investigated effect of simultaneous migration of coevolving bacteria and phages on the evolution of bacterial resistance and phage infectivity. Based on previous studies, that suggest that bacteria have a greater evolutionary potential than phages [17,23], we hypothesised that phages should benefit more from migration than bacteria. Our data were consistent with this hypothesis. Analysis of sympatric resistance revealed a negative linear effect of migration rate (Fig. 1; *F*_1,28 _= 6.65, *P *= 0.015), no quadratic effect of migration (*F*_1,28 _= 0.34, *P *= 0.2) and no significant difference between founding populations (*F*_5,28 _= 1.52, *P *= 0.2). However the relationship between migration rate and sympatric resistance was likely to be solely due to the high rates of sympatric resistance at 0% migration; indeed, when the 0% migration treatment was excluded from the analysis, we found no significant effects of migration rate on sympatric infectivity (Fig. 1; *P *> 0.2 for linear and quadratic effects). Thus any migration benefited the more genetically limited and so less evolvable phages more than bacteria, regardless of rate; and changing the rate did not increase or decrease its advantage.

These data suggest that simultaneous migration is likely to benefit the least evolvable species most during host-parasite antagonistic arms races. In the context of bacteria-phage coevolution, bacteria tend to be ahead in the arms race [23], and so simultaneous migration is more likely to favour phages. It is unclear whether simultaneous migration will generally benefit hosts or parasites most. Patterns of local adaptation in natural populations suggest either hosts or parasites may have an evolutionary advantage, and not always parasites as common wisdom suggests (reviewed in Kaltz & Shykoff [24]).

**Figure 2:**
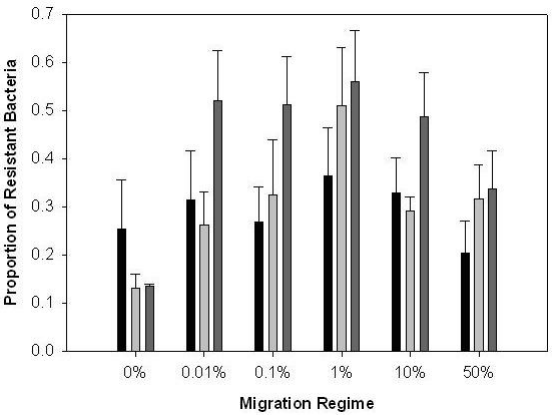
The effect of migration rate upon evolution of bacterial resistance ranges through time. Bars show mean (± 1 SEM) proportion of bacteria resistant to phages from all migration treatments from the same time point. Black bars are transfer (time point) 4; light grey bars, transfer 8; dark grey bars, transfer 12. The system is directional with bacteria becoming more resistant to a wider range of genotypes, so a higher proportion of resistant bacteria indicates that populations have evolved more rapidly.

We also addressed how migration rate affected global resistance of bacteria (i.e. the average resistance of bacteria from one treatment to phages from all other migration treatments) and global infectivity of phages (i.e. the average infectivity of phages from one treatment to bacteria from all other treatments), measuring infectivity and resistance across the range of migration regimes. We found a unimodal relationship between mean global bacterial resistance (to phages from all migration regimes) and the rate of migration, with resistance peaking at a migration rate of 1% per transfer (Fig. 2; quadratic effect of migration rate: *F*_1,28 _= 14.73, *P *= 0.001; there was no significant linear relationship between resistance and migration rate; linear effect of migration rate: *F*_1,28 _= 1.43, *P *= 0.241. Average levels of resistance were significantly different between replicates; effect of founding population: *F*_5,28 _= 2.82, *P *= 0.035). In this system, coevolution results in bacteria and phages evolving to be resistant and infective to an increasingly wide range of phage and bacterial genotypes, respectively [19,25]; a situation broadly consistent with GFGM of coevolution [20,26,27]. As such, initial increases in migration rate will provide genetic variation that will allow a given resistance range to be reached more rapidly. However, further increases in migration caused the rate of resistance evolution to decline (Fig. 2). This is likely to be because migration allowed a globally fit bacterial clone at a given point in time to spread rapidly through all populations, at the expense of clones with resistance alleles that might have been beneficial in the future. Specifically, we suggest that clones with alleles conferring resistance to a wider range of phages than is currently useful, increase in frequency in isolated tubes. However, because of pleiotropic growth rate costs associated with wide resistance ranges, high rates of migration would increase the probability of these clones being competitively excluded by clones with resistance ranges that are narrower but sufficient to resist contemporary phage genotypes (clonal interference; [28]). Consistent with this hypothesis, previous work on this bacterium suggests the operation of trade-off between resistance and competitive ability in the absence of phages [29]. Similar costs of resistance have been reported in other bacteria-phage systems (reviewed in Bohannan & Lenski [30]).

**Figure 3:**
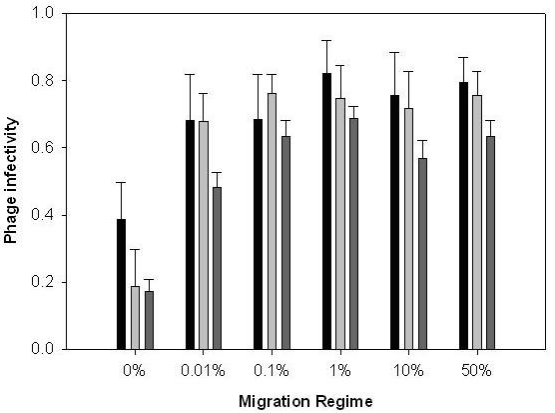
The effect of migration rate upon evolution of phage infectivity ranges through time. Bars show mean (± 1 SEM) proportion of bacteria from all migration treatments from the same time point that are sensitive to phages. Black bars are transfer (time point) 4; light grey bars, transfer 8; dark grey bars, transfer 12.

By contrast, phage infectivity ranges (measured against bacteria from all migration regimes) showed a positive relationship with migration rate (Fig. 3; linear effect of migration rate: *F*_1,28 _= 19.40, *P *< 0.001), which plateaued at around 1% migration (quadratic effect of migration rate: *F*_1,28 _= 12.44, *P *= 0.001). There was no effect of starting population (*F*_5,28 _= 1.98, *P *= 0.113). As with the analysis of sympatric resistance above, the relationship between phage infectivity and migration rate disappeared when the no migration treatment was excluded from the analysis (*P *> 0.1 for both linear and quadratic terms). These patterns of global resistance and infectivity with respect to different rates of migration are broadly consistent with patterns of sympatric resistance. Sympatric resistance decreases with migration, hence migration benefits phages more than bacteria. Low levels of migration appear to increase both global bacterial resistance and phage infectivity, whereas further increases in migration do not affect global phage infectivity but decrease global bacterial resistance. The net effect is that migration also appears to benefit phages more than bacteria when resistance and infectivity traits are measured across meta-populations.

There are two plausible explanations as to why bacterial resistance declined, but phage infectivity plateaued at high migration rates (compare Figs 2 and 3). The first possibility is (as above) that phages are simply less evolvable than bacteria. Specifically, the likelihood of beneficial alleles being lost as a result of clonal interference may have been less in phage than bacteria populations, because phage populations contained fewer competing beneficial mutations at any given time. Thus, beneficial mutations became fixed in phage populations prior to new mutations arising. Second, increased phage infectivity range may not be associated with the same growth rate costs associated with bacterial resistance ranges. As such, an allele that conferred a broader infectivity range than was currently necessary at a given time point may not have been outcompeted by alleles conferring less broad infectivity ranges. However, we have observed that phages that have coevolved with bacteria tend to produce much smaller plaque sizes than ancestral phages, suggesting there is also a cost to increased infectivity ranges.

It is initially surprising that the 0% and 50% migration treatments generated such different results: such high migration may simply have the effect of producing a single 3-fold larger population. However, resistance and infectivity traits readily evolve within a single transfer (7 generations), and it is likely that this evolution will be divergent between populations [19]. This will increase genetic variation within the metapopulation beyond a simple population size effect.

Our measures of sympatric resistance with respect to migration are likely to hold true through coevolutionary time: if migration benefits phages more, then sympatric infectivity is likely to be higher. However, the global data analysed here clearly represents a very specific and non-equilibrium coevolutionary state. Bacteria and phage evolve increasing resistance and infectivity through time, but such increases are likely end at some point [27]. As such, comparisons of resistance and infectivity ranges as a function of migration rates may only be relevant to this particular period of coevolution. However, it is possible that migration regimes may affect average levels of resistance and infectivity ranges (a dynamic equilibrium state) measured over much longer time scales. Escalatory arms races, as observed here, may represent an ascending phase of a coevolutionary cycle, with selection for narrow resistance and infectivity ranges as costs of resistance and infectivity become too great. [27]. The continual supply of 'good' resistance and infectivity traits through migration may, for example, increase the magnitude of escalation before this happens. That aside, our measures of resistance and infectivity may be more generally applicable if they are equated with the rate of evolution of coevolving populations of hosts and parasites. This may be a reasonable assumption when it is considered that resistance and infectivity ranges increases through time [19], and where specifically investigated, we observe that populations that coevolve faster show broader resistance ranges [25]. Thus, assuming that selection fluctuates to some degree through time (which it always will, unless coevolution is a pure GFGM and resistance and infectivity evolution is cost-free) during antagonistic coevolution, we predict unimodal relationships between the rate of evolution and migration rate when clonal interference can occur. By contrast, in the absence of clonal interference (and populations are very mutation limited), we expect this relationship to be positive.

## Conclusion

In summary, these results demonstrate that simultaneous migration of coevolving hosts and parasites can differentially affect their evolution, in this case benefiting the less evolveable (have a lower evolutionary potential) parasites more than hosts. Whether or not migration generally benefits the least evolvable coevolving partner remains to be seen. Either way, increased global movement of humans and agricultural produce and associated parasites may not only increase disease transmission, but also result in evolutionary change in levels of parasite infectivity and host resistance.

## Methods

### (a) Initiating Populations

This experiment looked at the effects of migration upon coevolution, more precisely how migration rate introduces variation to fuel coevolution. The starting populations for the migration treatments were initially transferred without migration to allow some initial differentiation between populations. Otherwise with no initial differentiation, between population migration will not bring in new variation. Eighteen replicate populations were initiated using approximately 10^7 ^cells of isogenic *Pseudomonas fluorescens *SBW25 and approximately 10^5 ^isogenic particles of phage SBW25Φ 2 [19]. Note that the minimal generation times of bacteria and phages are similar: approximately 40 minutes. Cultures were grown in static 30 ml glass universals with loose plastic caps containing 6 ml of King's Media B, grown at 28°C. Every 48 hours 60 μl of culture was transferred to a fresh microcosm. Cultures were regularly frozen in 20% glycerol at -86°C for long-term storage. After six transfers, each population was used to seed six new replicate tubes, each of which was assigned to one of 6 migration treatments, resulting in a total of 108 (18 * 6) tubes. The eighteen tubes within each treatment were assigned to one of six metapopulations, each containing 3 tubes. Note that the same 3-tube combinations were used in each treatment. Migration was carried out within each metapopulation, resulting in 6 independent replicates within each treatment.

### (b) Migration Regimes

Cultures were propagated for a further twelve transfers but exposed to one of the six following treatments immediately prior to transfer: Control (no migration), 0.01% migration, 0.1% migration, 1% migration, 10% migration and 50% migration. To migrate cultures, the specified percentage of culture was removed from each of the three microcosms within a replicate to a common pool and mixed. The same volume of culture added to the common pool from each replicate was then transferred from the common pool to each microcosm within the replicate [17]. Every fourth transfer cultures were frozen in 20% glycerol at -86°C and samples of phages were isolated from bacteria by vortexing 900 μl of culture with 100 μl of chloroform, followed by 2 minutes centrifugation at 13000 rpm. This lysed and pelleted bacteria, leaving phages in the supernatant. These samples of phages were stored at 4°C.

### (c) Resistance/Infectivity Assays

Bacteria were plated out on to King's Agar B from the frozen stocks, and ten random independent colonies from each population were streaked out to make stock plates. The stock plates were confirmed to be free from phages after visual examination, as no plaques were observed. A sample of phages (20 μl; 0.33% of the total population) was streaked on to a King's agar B plate, allowed to dry, and the ten independent bacterial colonies were then streaked perpendicularly across the line. Plates were incubated at 28°C for 24 hours prior to examination. A bacterial colony was classed as sensitive to a phage population if there was any inhibition of growth (as determined by eye), otherwise it was classed as resistant [17,19,25,29].

We determined the proportion of resistant bacteria from a single tube within each 3-tube metapopulation to phages from their own tube (to determine sympatric resistance), and then from single tubes from each of the other migration treatments (to determine average global resistance). Tubes from other migration treatments were chosen on the basis of shared founding populations: for example, replicate 1 in the no migration shared the same founding population as replicate 1 in the 0.01%, 0.1%, 1%, 10%, and 50% migration treatments, etc. The infectivity of a single phage population per metapopulation (global infectivity) was assayed in the same way, with infectivity defined as the proportion of sensitive bacteria. This assay was carried out at transfers four, eight, and twelve.

### (d) Statistical Analyses

Sympatric resistance is defined as the proportion of bacteria resistant to phages from the same tube, and, mean sympatric resistance through time was analysed using a General Linear Model (GLMs) carried out in MINITAB, fitting log10 (treatment + 0.01) as both a linear and quadratic covariate and founding population as a factor. Founding population was treated as a random factor, although the lack of either nesting or replication for the line-by-treatment interaction in the study, means that error MS is used as the denominator for calculating *F*-ratios in all cases [31]. In other words, line was treated in the analyses as a fixed effect. To meet GLM assumptions of normality, linearity and homogeneity of variance, phage infectivity was squared [32]. Sympatric resistance was log10-transformed to meet GLM assumptions.

We then calculated the mean proportional resistance of each bacterial population to all phages against which it was assayed, to provide average measures of resistance across all migration regimes. Similarly, we calculated the mean infectivity (1-proportion resistant bacteria) of each assayed phage population. Data were analysed as above, but no transformations were required.

## Authors' contributions

ADM contributed to the conception of the experiment, experimental work, analyses and drafting of the manuscript. MAB and LLP contributed to the experimental work. CP provided theoretical advice. AB contributed to the conception of the experiment, analyses and drafting of the manuscript. All authors contributed to the final manuscript preparation.
